# Prion strains depend on different endocytic routes for productive infection

**DOI:** 10.1038/s41598-017-07260-2

**Published:** 2017-07-31

**Authors:** Andrea Fehlinger, Hanna Wolf, André Hossinger, Yvonne Duernberger, Catharina Pleschka, Katrin Riemschoss, Shu Liu, Romina Bester, Lydia Paulsen, Suzette A. Priola, Martin H. Groschup, Hermann M. Schätzl, Ina M. Vorberg

**Affiliations:** 1 Deutsches Zentrum für Neurodegenerative Erkrankungen e.V., Sigmund-Freud-Strasse 27, 53127 Bonn, Germany; 2 Institut für Virologie, Technische Universität München, Trogerstr. 30, 81675 München, Germany; 3 Laboratory of Persistent Viral Diseases, Rocky Mountain Laboratories, National Institute of Allergy and Infectious Diseases, National Institutes of Health, 903 South 4th Street, Hamilton, MT 59840 USA; 4 Friedrich-Loeffler-Institut, Institute of Novel and Emerging Infectious Diseases, 17493 Greifswald-Insel Riems, Germany; 5 Dept. of Comparative Biology & Experimental Medicine, University of Calgary, Calgary, AB T2N 4Z6 Canada; 6 Department of Neurology, Rheinische Friedrich-Wilhelms-Universität, 53127 Bonn, Germany

## Abstract

Prions are unconventional agents composed of misfolded prion protein that cause fatal neurodegenerative diseases in mammals. Prion strains induce specific neuropathological changes in selected brain areas. The mechanism of strain-specific cell tropism is unknown. We hypothesised that prion strains rely on different endocytic routes to invade and replicate within their target cells. Using prion permissive cells, we determined how impairment of endocytosis affects productive infection by prion strains 22L and RML. We demonstrate that early and late stages of prion infection are differentially sensitive to perturbation of clathrin- and caveolin-mediated endocytosis. Manipulation of canonical endocytic pathways only slightly influenced prion uptake. However, blocking the same routes had drastic strain-specific consequences on the establishment of infection. Our data argue that prion strains use different endocytic pathways for infection and suggest that cell type-dependent differences in prion uptake could contribute to host cell tropism.

## Introduction

Prions are proteinacious infectious agents that cause transmissible spongiform encephalopathies, fatal neurological disorders of mammals^1^. A misfolded, aggregated isoform (PrP^Sc^) of the cellular prion protein (PrP^C^) is the major if not sole component of the infectious agent^2, 3^. In the central nervous system, prion deposition has been observed in association with neurons, astrocytes and microglia as well as ependymal or endothelial cells^4–6^. PrP^Sc^ molecules replicate by binding to PrP^C^ and templating its conversion into an infectious isoform. PrP^C^ is a conserved cell surface glycoprotein that resides within cholesterol- and sphingolipid-enriched cell surface microdomains, such as caveolae or lipid rafts^7–10^. PrP^Sc^ formation occurs on the cell surface and/or within intracellular vesicles following internalisation of prion particles^11–17^. The exact site(s) of prion replication and the cellular events that lead to productive infection have not been resolved.

Prions exist as strains with specific biological and biochemical properties^18^. In rodents with experimental prion disease, strains can be discriminated by incubation times, clinical signs and neuropathological features^18^. Prion strains preferentially target specific brain regions and cause characteristic lesion profiles and PrP^Sc^ deposition patterns. Prion strains differ in their cell tropism, with some strains exhibiting high tropism for astrocytes and low tropism for neurons and vice versa^19^. As prions lack coding nucleid acid, strain-specific information cannot be encoded within genes. Instead, differences in the high-order structure of PrP^Sc^ multimers are proposed to encipher heritable strain information^20^. How exactly the conformational diversity of PrP^Sc^ multimers associated with different strains relates to different disease phenotypes is unknown.

One possible explanation for cell tropism could be that strains use different cellular receptors or require different cofactors for efficient replication. While several putative prion receptors have been proposed, their roles as general prion receptors are unclear^21–24^. Likewise, elegant studies over the last years have identified endogenous cofactors such as phospholipids or polyanions that promote replication of certain prion strains *in vitro* ^25, 26^. It has been speculated that prion strains depend on different sets of so far unknown cofactors^27^. Curiously, certain cell lines can faithfully propagate different prion strains, while other cell lines are permissive to only one strain^28, 29^. Thus, it is possible that some cell lines lack cellular factors required by certain prion strains. Alternatively, cofactors might be confined to cellular sites to which the specific prion strain has only limited access^30^. Restricted access to certain subcellular compartments could be the result of different uptake mechanisms for different prion strains. It has been shown previously that the route of internalisation can have drastic consequences on the fate of a pathogen and its ability to establish infection^31, 32^. We therefore hypothesised that prion strains use different cellular trafficking pathways for cell invasion and productive infection.

Unfortunately, studying cellular aspects of prion replication has been notoriously difficult due to the low number of permissive cell lines and their restricted susceptibility to only certain prion strains (for a review, see ref. 33). Even in susceptible cell populations, only a limited number of cells become infected^34^. The difficulty to discriminate PrP isoforms as well as exogenous from *de novo* generated abnormal PrP further restricts detailed microscopic analysis of prion replication. Substantial efforts over the last years led to the isolation of cell sublines with increased susceptibility^24, 35–37^. Some of the most widely used cell lines in prion research to date are of non-neuronal origin, including a growing number of fibroblast cell lines^28, 29, 36–40^ and primary fibroblasts^41^. Importantly, recent evidence accumulates that fibroblast-like cells constitute targets of prions *in vivo* ^42, 43^. The peculiar susceptibility of fibroblasts to different prion strains makes them particularly suitable for elucidating basic principles of prion strain replication that so far have been unexplored.

In the present study, we aimed to answer if prion strains that are able to replicate in the same cell population might use different internalization pathways to establish persistent infection. Different requirements for specific endocytosis pathways could indicate that the subcellular compartments involved in establishing productive infections differ for different strains. To this end we examined the role of canonical endocytic pathways implicated in prion uptake and establishment of chronic prion infection. A L929 mouse fibroblast cell subline was chosen based on its high susceptibility to prion strains 22L and RML^24, 28^. Our data reveal 1) that blockage of main endocytic routes does not drastically affect prion entry, 2) that manipulation of endocytosis differentially affects the establishment of infection by different prion strains and 3) that endocytic pathways play different roles during early and late stages of prion infection. The fact that prion strains are differentially sensitive to perturbations in endocytosis during the infection process argues that prion strains rely on different endocytic routes for productive infection. This finding supports the hypothesis that selective trafficking of prion strains through specific subcellular compartments controls access to the cofactors or microenvironments required for prion replication.

## Results

### PrP^C^ in prion-permissive L929 cells localizes to the cell surface, associates with detergent-resistant membranes and is endocytosed *via* clathrin-mediated endocytosis

Aim of this study was to test the hypothesis that different prion strains depend on different internalisation routes and might therefore differ in their requirements for subcellular compartments involved in the establishment of infection. To this end, a cell line highly susceptible to different prion strains that is amenable to manipulation of endocytosis pathways was required. We chose to perform our analysis using a murine fibroblast cell line susceptible to different prion strains. L929 cells are a well established cell culture model in prion research and have been fundamental in elucidating basic principles in prion biology^24, 29, 40, 44^. A clone (L929 - 15.9) of L929 mouse fibroblasts^44^ was selected based on its high susceptibility to mouse-adapted prion strains 22L and RML (Supplementary Table). Previous studies have demonstrated that caveolae or lipid rafts as well as clathrin-mediated endocytosis (CME) are involved in the internalization of PrP^C^ and might also play a role in prion replication^8, 45–47^. Caveolae are a special class of rafts highly enriched in caveolins, integral membrane proteins that regulate trafficking and sorting of caveolae. Caveolin-1 (Cav-1) expression is required for caveolar biogenesis^48^, while clathrin-coated pits are characterised by the coat protein clathrin and mediate uptake of a diverse set of molecules from the extracellular space^49^. Both clathrin- and caveolin-mediated endocytosis pathways have been successfully manipulated in L929 cells to study uptake of viruses^50^.

**Figure 1:**
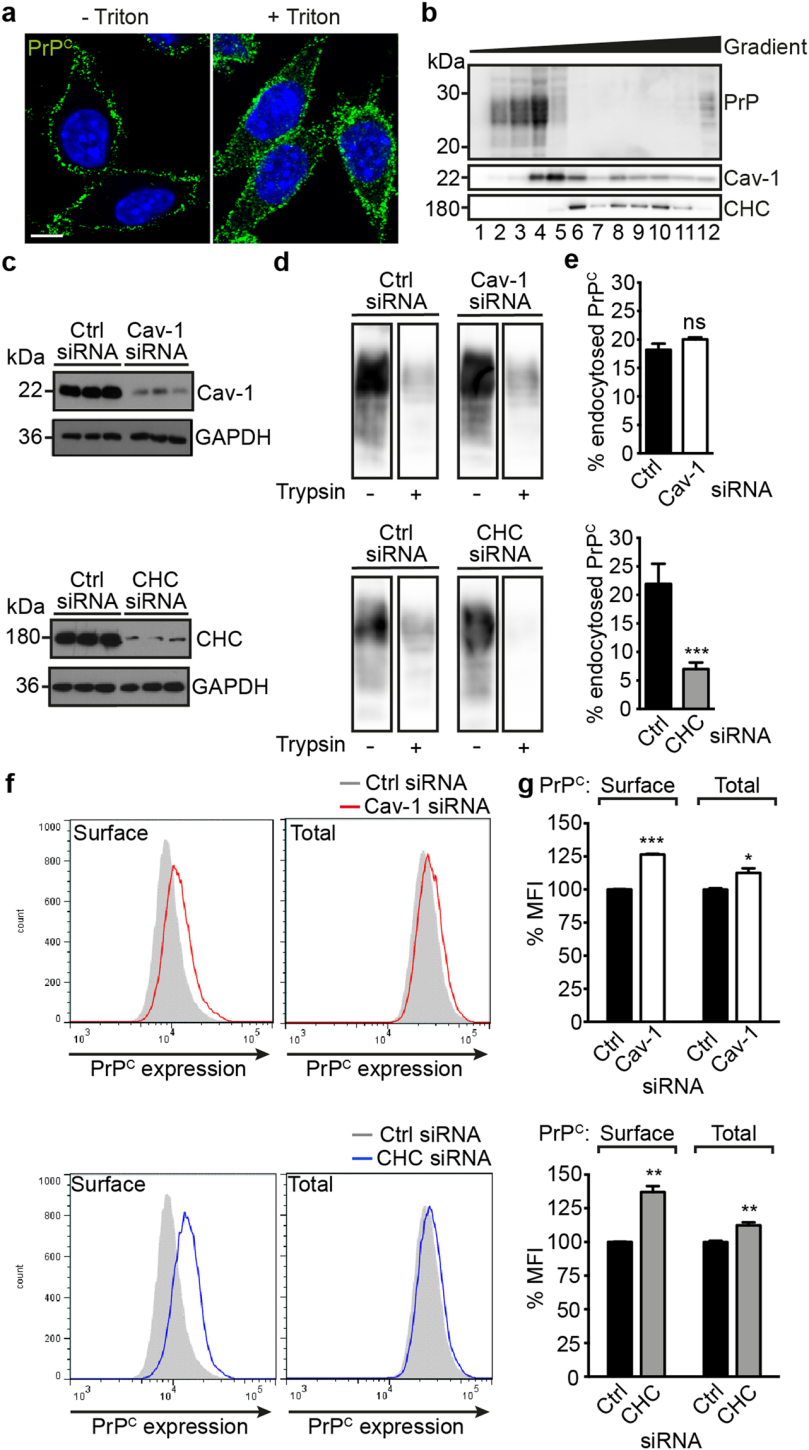
Localization and internalization of cellular PrP in L929 fibroblast cells. (**a**) Confocal microscopy analysis of non-permeabilized (−Triton) and permeabilized (+Triton) L929 cells. PrP^C^ was detected using monoclonal anti-PrP antibody 4H11 (green), nuclei were stained with Hoechst (blue). Scale bar = 5 μm. (**b**) PrP^C^ and Cav-1 partially co-localize in detergent-resistant membrane fractions. Lysates from L929 - 15.9 cells were applied to sucrose gradients and fractions were analyzed by western blot. (**c**) Knock-down of CHC and Cav-1 was determined by western blot analysis of cell lysates harvested 72 h post transfection. GAPDH serves as a loading control. (**d**) Cell surface biotinylation of L929 cells 72 h post transfection with Cav-1 or CHC siRNAs. Cells were chased at 37 °C for 60 min to allow PrP^C^ internalization and either incubated with Trypsin (+) to remove cell surface proteins or left untreated (−) for evaluation of total biotinylated PrP^C^. PrP^C^ in cell lysates was immunoprecipitated with mAb 4H11 and biotinylated PrP was detected using horseradish peroxidase- (HRP-) conjugated streptavidin. Bands originate from the same western blot (same acquisition settings). Additional lanes were excized for presentation purposes. (**e**) Quantification of the biotinylation assay displayed in (**d**). The amount of biotinylated internalized PrP^C^ (+Trypsin in **d**) was expressed as the percentage of total biotinylated PrP^C^ detected in control cells not treated with Trypsin (−Trypsin in **d**). The mean of the control cells was set to 100%. Bars represent mean values ± SD. Signals of three independent transfections/biotinylations were quantified. Statistics were performed using unpaired two-tailed t test. (***p < 0.001, ns = not significant). (**f**) Flow cytometry analysis of PrP^C^ levels after siRNA-mediated knock-down of Cav-1 or CHC 72 h post transfection. Cells were either permeabilized to detect total PrP (right panels) or left intact for detection of cell surface PrP (left panels). (**g**) Measured mean fluorescence intensities (MFIs) in (**f**) were normalized to the mean of control cells set to 100%. Three independently transfected cell populations for each siRNA were measured. Bars represent mean values ± SD. Statistical analysis was performed using the unpaired two-tailed t test. Significant changes are indicated by asterisks (*p < 0.05; **p < 0.01; ***p < 0.001).

In several cell lines, PrP^C^ localizes to rafts or caveolae prior to its internalization by CME^23, 51, 52^. We first determined the endocytic route of PrP^C^ in L929 cells. In uninfected L929 cells, PrP^C^ was located on the cell surface and within small puncta in the cytosol (Fig. 1a). PrP^C^ partially segregated with sucrose gradient fractions harboring Cav-1, but not with fractions containing clathrin heavy chain (CHC) (Fig. 1b), suggesting that in L929 fibroblasts, PrP^C^ is localized in caveolae. Small interfering RNA (siRNA) was used to disrupt either CME or caveolae-mediated endocytosis. As expected, silencing CHC or Cav-1 expression (Fig. 1c) functionally impaired CME and caveolae-mediated endocytosis of fluorescently labelled transferrin or choleratoxin, respectively (Supplementary Fig. S1). Knock-down of CHC also significantly decreased the uptake of biotinylated cell surface PrP^C^ (Fig. 1d, e, lower panel). No decrease in internalized PrP^C^ was observed when Cav-1 was downregulated (Fig. 1d, e, upper panel). Thus, PrP^C^ in L929 - 15.9 cells partially localizes in membrane environments enriched in Cav-1 and is preferentially endocytosed *via* clathrin-coated pits. Interestingly, both Cav-1 and CHC knock-down slightly increased total and cell surface expression levels of PrP^C^ (Fig. 1f, g). This suggests that caveolae and clathrin might cooperate in internalizing PrP^C^ ^45, 53^.

### PrP^Sc^ accumulation in persistently infected cells depends on Cav-1 expression

**Figure 2:**
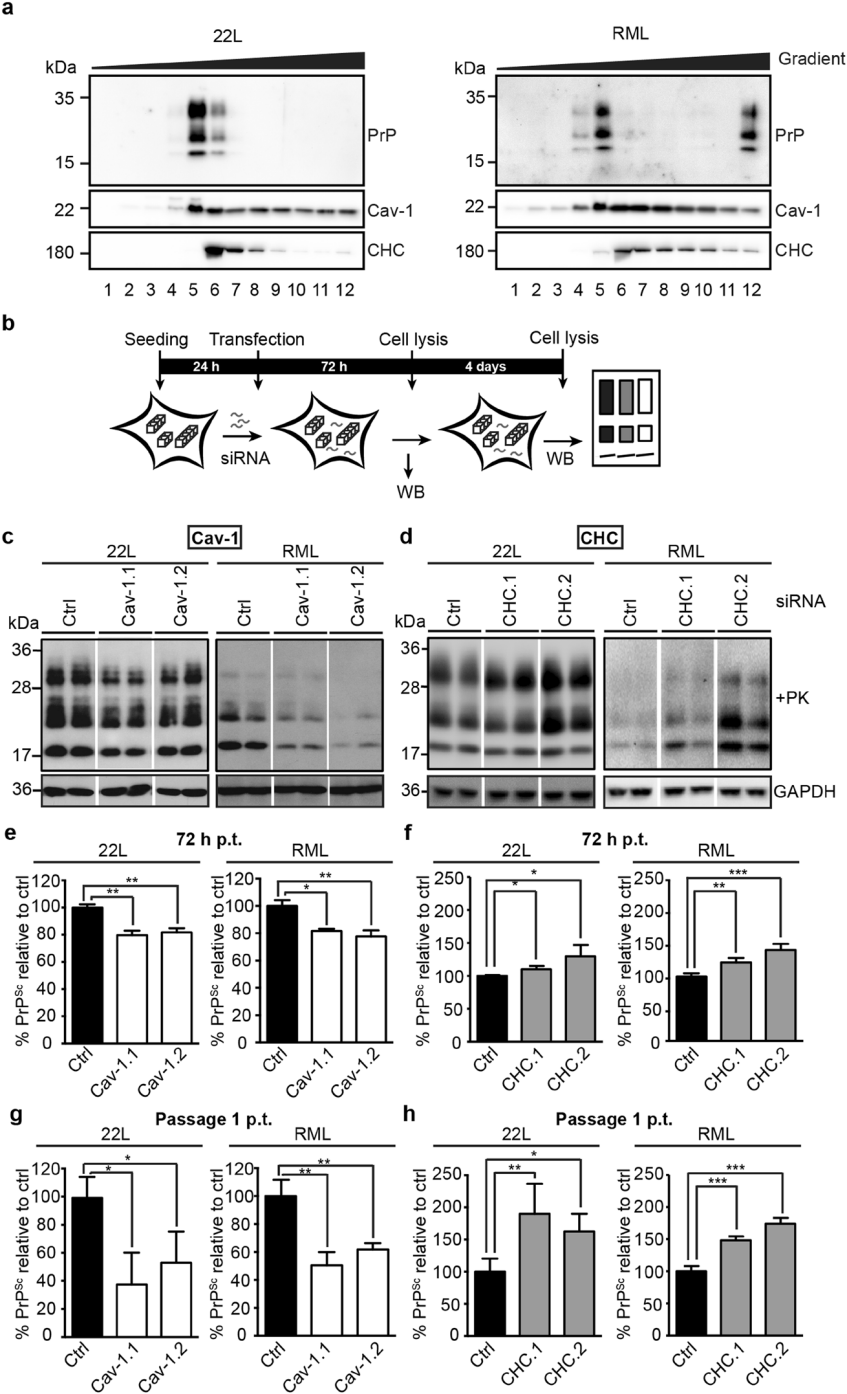
Caveolae are required for persistent PrP^Sc^ accumulation in L929 cells. (**a**) Sucrose gradient fractionation of cleared cell lysates of L929^22L^ and L929^RML^ cells. Proteins in gradient fractions were subjected to proteinase K(PK) (50 μg/ml, 45 min, 37 °C) and subsequently analyzed by western blot using mAb 4H11. Shown are representative blots of three fractionated lysates per cell line. (**b**) Experimental setup for transient siRNA transfection of persistently infected L929 cells. L929 cells chronically infected with 22L or RML prions were transfected with either non-silencing siRNA or with two siRNAs against Cav-1 (Cav-1.1, Cav-1.2) or CHC (CHC.1 and CHC.2). (**c**) L929^22L^ or L929^RML^ cells transfected with siRNAs against Cav-1 or non-silencing control siRNA (Ctrl) were tested for PrP^Sc^ content (+PK) after PK treatment by western blot 72 h post transfection (p.t.). GAPDH protein levels demonstrate comparable protein levels in the non-PK treated samples. For presentation purposes additional lanes were excised. (**d**) Same experimental setup as shown in (**c**) but with siRNA against CHC. (**e**,**f**) Statistical analysis of PrP^Sc^ signals 72 h post transfection with siRNA against Cav-1 (**e**) or CHC (**f**). The mean signal value of the control was set to 100%. Bars represent mean values ± SD. Statistical analysis was performed using one-way ANOVA with Dunnett’s multiple comparisons test. For each siRNA, three independent transfections were performed (*p < 0.05, **p < 0.01, ***p < 0.001). Experiments were repeated at least twice with similar results. (**g**,**h**) Statistical analysis of PrP^Sc^ signals passage 1 post transfection with siRNA against Cav-1 (**g**) or CHC (**h**) performed as above.

Next, we studied the role of caveolae-mediated endocytosis and CME on prion accumulation in chronically infected cells^44^. Subclone L929 15.9 persistently propagates prions when infected with prion strains 22L or RML (Suppl. Table). To test if PrP^Sc^ produced by cells chronically infected with either 22L (L929^22L^) or RML (L929^RML^) partitioned into similar detergent rich membranes like PrP^C^, we performed sucrose gradient density fractionation (Fig. 2a). Independent of the scrapie strain, PrP^Sc^ was recovered from low buoyant density fractions (fractions 4–6) that partially overlapped with Cav-1. Some overlap was also observed with CHC. Interestingly, RML PrP^Sc^ but not 22L PrP^Sc^ also segregated with very high density fractions. The difference in PrP^Sc^ partitioning with different sucrose gradient fractions suggests that the intracellular distribution of 22L and RML PrP^Sc^ slightly differs in L929 cells.

To study the influence of caveolae-mediated endocytosis or CME on the accumulation of PrP^Sc^ in persistently infected cells, levels of PrP^Sc^ were determined 72 h post transfection with siRNAs targeting both pathways (Fig. 2b). We chose two independent siRNAs for CHC and Cav-1 that resulted in knock-down efficiencies of at least 69.7% depending on the siRNA (Supplementary Fig. S2). Knock-down of Cav-1 expression significantly decreased PrP^Sc^ levels in both 22L or RML infected cells 72 h post transfection (Fig. 2c, e). Likewise, treatment of L929^22L^ or L929^RML^ cells with tyrosine kinase inhibitor Genistein, known to disrupt caveolae-dependent endocytosis, decreased the amount of PrP^Sc^ in 22L or RML infected cells in a dose-dependent manner (Supplementary Fig. S3). Knock-down of CHC had the opposite effect on PrP^Sc^ accumulation, leading to a slight increase of PrP^Sc^ 72 h post transfection (Fig. 2d, f). Several days post transfection, the effects of Cav-1 and CHC siRNA on PrP^Sc^ were even more pronounced (Fig. 2g, h). In conclusion, caveolae rather than CME were required for efficient PrP^Sc^ accumulation in chronically infected L929 cells.

### Caveolin-1-, clathrin- and dynamin-2-independent uptake of PrP^Sc^

**Figure 3:**
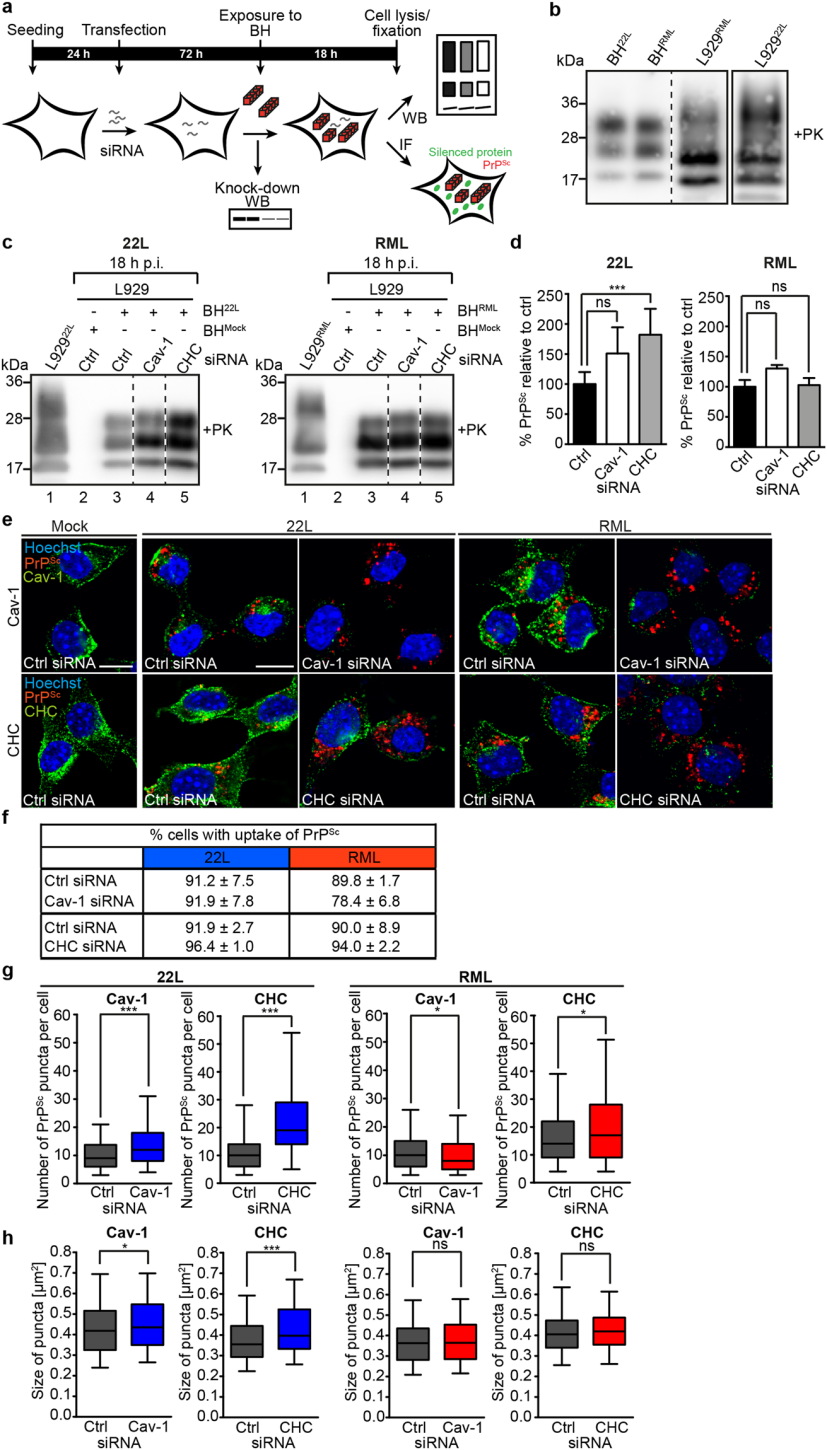
PrP^Sc^ is taken up by clathrin-, caveolin- and dynamin-independent routes. (**a**) Graphical illustration of the experimental setup. BH: Brain homogenate, WB: Western blot, IF: Immunofluorescence staining. (**b**) Comparison of brain homogenate PrP^Sc^ and PrP^Sc^ propagating in persistently infected L929 cells. Additional lanes were excised for presentation purposes. Sample L929^22L^ was run on the same gel but had a different exposure time to avoid oversaturation. BH: Brain homogenate. Anti-PrP antibody 4H11. (**c**) PrP^Sc^ bound to or internalized by siRNA-transfected L929 cells 18 h post exposure to 22L or RML prions. As controls, cell lysates of L929 cells chronically infected with either 22L or RML were loaded, demonstrating different sizes and glycosylation patterns characteristic for cell culture derived PrP^Sc^. For presentation purposes additional lanes were excised (dashed lines). PK: Proteinase K. (**d**) Statistical analysis of western blots shown in (**c**) was performed using one-way ANOVA with Dunnett’s multiple comparisons test (n = 3 biological replicates; ***p < 0.001, ns = not significant). (**e**) Uptake of 22L or RML PrP^Sc^ in siRNA transfected cells was monitored by immunoflourescence staining 18 h post exposure to prions. PrP^Sc^ was detected with mAb 4H11 (red) after denaturation with GdnHCl. Cav-1 and CHC were detected with the appropriate pAbs (green). Nuclei were stained with Hoechst (blue). Cells exposed to Mock brain homogenate served as controls. Every sample group was imaged with identical settings. Scale bar: 10 µm. (**f**) Analysis of cells with PrP^Sc^ signal. A total number of at least 100 cells was scored. (**g**,**h**) Box plot demonstrating number and size of PrP^Sc^ puncta in cells exposed to 22L (blue) and RML (red) prions upon Cav-1 or CHC siRNA transfection. Plotted is the median with whiskers displaying the 5–95% percentile. Statistical analysis was performed using the non-parametric Mann-Whitney test. Asterisks indicate significant differences between cells treated with non-silencing siRNA and siRNA directed against Cav-1 or CHC (*p < 0.05, ***p < 0.001, ns = not significant).

Early events in prion biogenesis are binding of exogenous PrP^Sc^ to the cell surface and subsequent PrP^Sc^ uptake into intracellular compartments^24, 54^. Association of PrP^Sc^ with the cells was first assessed by western blot analysis 18 h post exposure to prion strains (Fig. 3a–d). The PrP^Sc^ signal detected in the scrapie brain homogenate treated cell populations likely represents PrP^Sc^ originating from the inoculum, as it markedly differed in size and glycosylation pattern from that of cells persistently infected with prion strains 22L or RML (Fig. 3b,c). Control experiments using L929 cells expressing 3F4 antibody epitope-tagged mouse PrP confirmed that 18 h post exposure, *de novo* generated PrP^Sc^ was not detectable (Supplementary Fig. S4). Substantial amounts of PrP^Sc^ were associated with cells in which Cav-1 or CHC were downregulated by siRNA (Fig. 3c). Silencing of CHC expression during 22L prion exposure led to an almost two-fold increase in cell-associated PrP^Sc^, while no significant increase in PrP^Sc^ was observed when cells were silenced for Cav-1 (Fig. 3d, left panels). By contrast, knock-down of Cav-1 or CHC did not significantly affect the amount of cell-associated PrP^Sc^ when cells were exposed to RML prions (Fig. 3d, right panels).

We performed antigen denaturation^55, 56^ and indirect immunofluorescence to assess the amount of internalized PrP^Sc^ following exposure to prion brain homogenate. As a control, we first tested if PrP^C^ might serve as an uptake receptor for PrP^Sc^. In line with previous studies^33, 54, 57, 58^, expression of PrP^C^ was not required for PrP^Sc^ internalization, suggesting that prion uptake was independent of PrP^C^ (Supplementary Fig. S5). By contrast, upon knock-down of Cav-1, L929 cells exhibited increased internal 22L PrP^Sc^ staining compared to cells transfected with non-silencing siRNA (Fig. 3e). High levels of intracellular 22L PrP^Sc^ staining were also apparent when cells were transfected with siRNA directed against CHC (Fig. 3e). Similarly, substantial amounts of RML PrP^Sc^ were taken up by cells silenced for Cav-1 or CHC expression, arguing that these endocytic pathways are not essential for PrP^Sc^ uptake (Fig. 3e). Silencing of Cav-1 slightly reduced the number of cells that had taken up RML PrP^Sc^, while the same treatment left the uptake of 22L PrP^Sc^ unaffected (Fig. 3f). Still, PrP^Sc^ nternalization was generally highly efficient, leading to more than 70% of positive cells (Fig. 3f). Impairment of endocytic pathways influenced cellular distribution of PrP^Sc^ puncta (Fig. 3g, h). Quantitative image analysis revealed that Cav-1 and CHC siRNA transfection increased both number and size of 22L PrP^Sc^ puncta compared to control cells (Fig. 3g, h, blue box plots). By contrast, Cav-1 silencing slightly reduced the number of RML PrP^Sc^ puncta but left the size of puncta unaffected (Fig. 3f, g, red box plots, left panels). CHC silencing increased the number but not size of RML PrP^Sc^ puncta (Fig. 3g, h, red box plots, right panels).

The foregoing experiment suggested that 22L and RML prions were taken up predominately by clathrin- and caveolin-independent pathways, or that the two strains can be internalized by either one of the pathways, dependent on accessibility. Attempts to downregulate Cav-1 and CHC simultaneously were unsuccessful due to toxic effects of the siRNA treatments (data not shown). Therefore, we tested if functional impairment of dynamin-2 downstream of clathrin and Cav-1 prevents PrP^Sc^ internalization (Supplementary Fig. S6). Expression of the dominant negative dynamin-2 (Dyn K44A), a mutant that selectively and effectively inhibits dynamin-dependent endocytic pathways including both CME and caveolin-dependent uptake^59, 60^ (Supplementary Fig. S6a, b), was unable to block PrP^Sc^ uptake (Supplementary Fig. S6c, d). Thus, PrP^Sc^ predominantly enters L929 cells by pathways independent of clathrin-, caveolin-1 and dynamin-2, or is able to bypass these routes when impaired. The finding that different strains were differentially sensitive to manipulations of endocytic routes suggests that slight differences in the uptake mechanisms of different strains exist.

### A fraction of PrP^Sc^ is internalized by macropinocytosis

**Figure 4:**
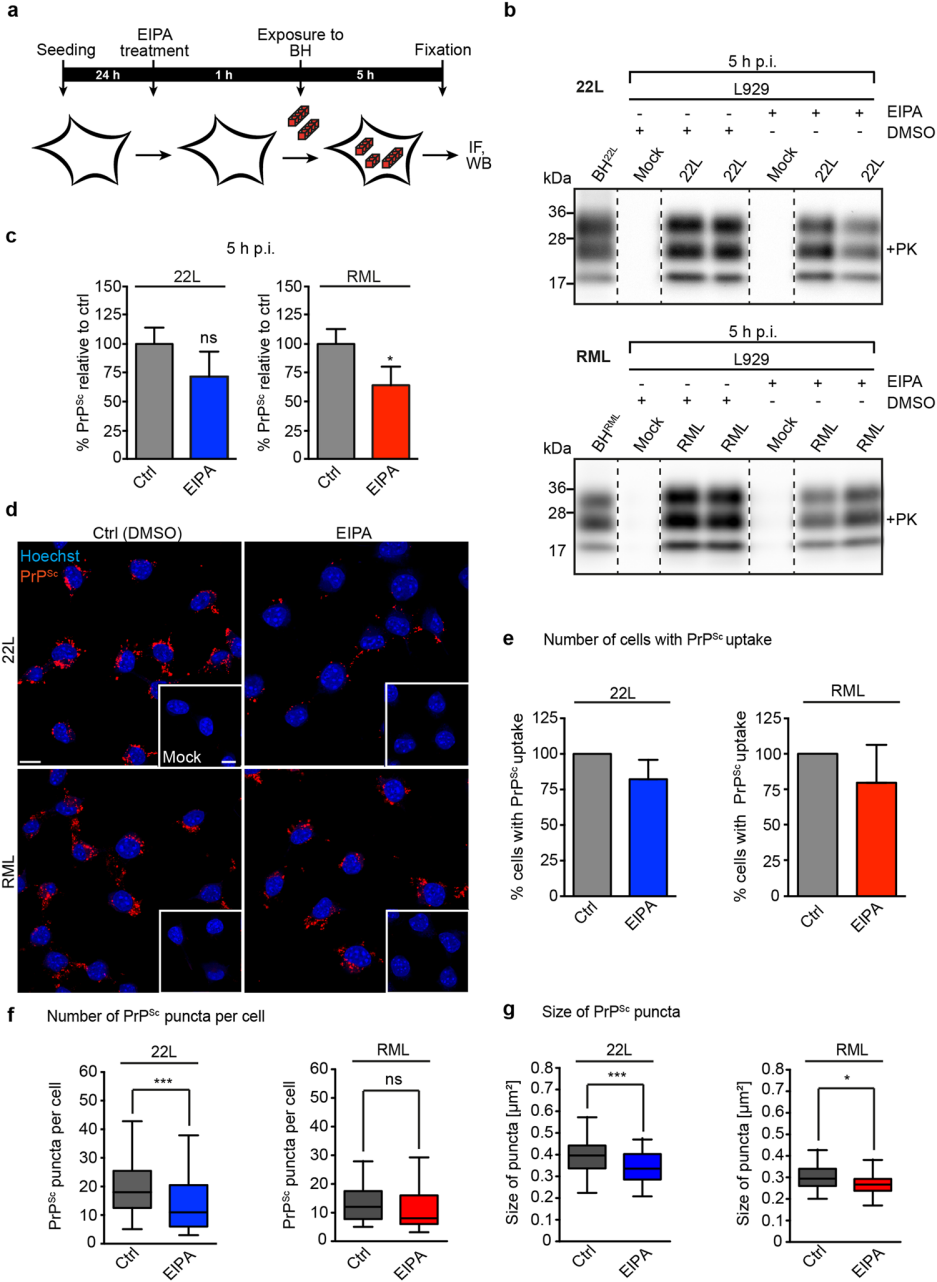
Effect of macropinocytosis inhibition on PrP^Sc^ uptake. (**a**) Experimental setup to study the effect of macropinocytosis inhibition on the uptake of PrP^Sc^. L929 cells were treated with EIPA for 1 h prior to exposure to brain homogenates (BH). Note that due to toxic effects of the inhibitor upon prolonged incubation, PrP^Sc^ was detected 5 h post exposure to BH. (**b**) PrP^Sc^ bound to or internalized by EIPA treated L929 cells 5 h post exposure to 22L or RML prions. As controls, BH from 22L or RML infected mice were loaded. For presentation purposes, additional lanes were excized (dashed lines). PK: Proteinase K. (**c**) Statistical analyses of western blots shown in (**b**) were performed using t test (n = 3 biological replicates; *p < 0.05, ns = not significant). (**d**) PrP^Sc^ was detected by immunofluorescence staining using mAb 4H11 (red). Cells were analyzed by confocal microscopy with identical imaging settings. Under the conditions used, PrP^C^ was not detected in Mock treated cells (insets). Nuclei were stained with Hoechst (blue). Scale bar: 10 µm. (**e**) Quantification of the relative percentages of cells that internalized 22L (blue) or RML (red) PrP^Sc^ when macropinocytosis was inhibited. Numbers were normalized to the mean of PrP^Sc^ positive cells in the siRNA controls set to 100%. Displayed are mean values ± SD (n = 3 biological replicates). (**f**,**g**) Quantification of the number and sizes of intracellular PrP^Sc^ puncta with and without EIPA treatment. Shown are box plots of the number (**f**) or sizes (**g**) of 22L (blue) or RML (red) PrP^Sc^ puncta. Plotted is the median with whiskers displaying the 5–95% percentile. Asterisks indicate significant changes between control and EIPA treated cells (*p < 0.05, ***p < 0.001, ns = not significant).

The fact that CHC silencing increased PrP^Sc^ uptake prompted us to test if alternative internalization routes might be upregulated by this treatment. Macropinocytosis is a relatively non-selective, clathrin- and caveolin-independent uptake route utilised by several pathogens^49, 61^ and potentially also prions in some cellular models^62^. Control experiments confirmed that chemical inhibition of macropinocytosis by EIPA reduced the uptake of macropinocytosis reporter FITC-dextran by 50% (Supplementary Fig. S7a, b). Interestingly, siRNA-mediated knock-down of CHC significantly increased the uptake of FITC-dextran, suggesting that internalization by macropinocytosis is enhanced when CME is impaired (Supplementary Fig. S7b). Thus, the increased uptake of 22L PrP^Sc^ upon CHC siRNA transfection could be due to increased macropinocytosis rates. By contrast, knock-down of Cav-1 had no significant effect on FITC-dextran uptake (Supplementary Fig. S7b). To study if macropinocytosis is involved in PrP^Sc^ uptake, L929 cells were treated with macropinocytosis inhibitor EIPA prior to exposure to brain homogenates (Fig. 4a). EIPA treatment slightly inhibited the association of RML PrP^Sc^ with L929 cells as assessed by western blot (Fig. 4b,c). No significant difference was observed for 22L. Internalized PrP^Sc^ was subsequently detected by immunofluorescence staining (Fig. 4d). EIPA treatment only slightly affected the number of cells that had taken up 22L or RML PrP^Sc^ (Fig. 4e), but significantly reduced number (Fig. 4f) and size (Fig. 4g) of 22L PrP^Sc^ puncta per cell. The same treatment had no effect on the number of RML PrP^Sc^ puncta per cell (Fig. 4f), but slightly reduced the size of RML PrP^Sc^ puncta (Fig. 4g). These results suggest that the increased uptake of 22L PrP^Sc^ upon CHC knock-down is likely due to increased macropinocytosis. As CHC downregulation and macropinocytosis inhibitor EIPA had only small effects on RML PrP^Sc^ uptake, macropinocytosis is not essential for cell invasion by the RML scrapie strain. Importantly, however, the fact that inhibition of three major uptake routes did not drastically impair PrP^Sc^ uptake strongly argues that prions can enter cells by multiple pathways, as has been documented for other pathogens^63^. Alternatively, additional endocytic routes might be involved in prion uptake.

### The establishment of productive prion infections involves different routes of endocytosis

**Figure 5:**
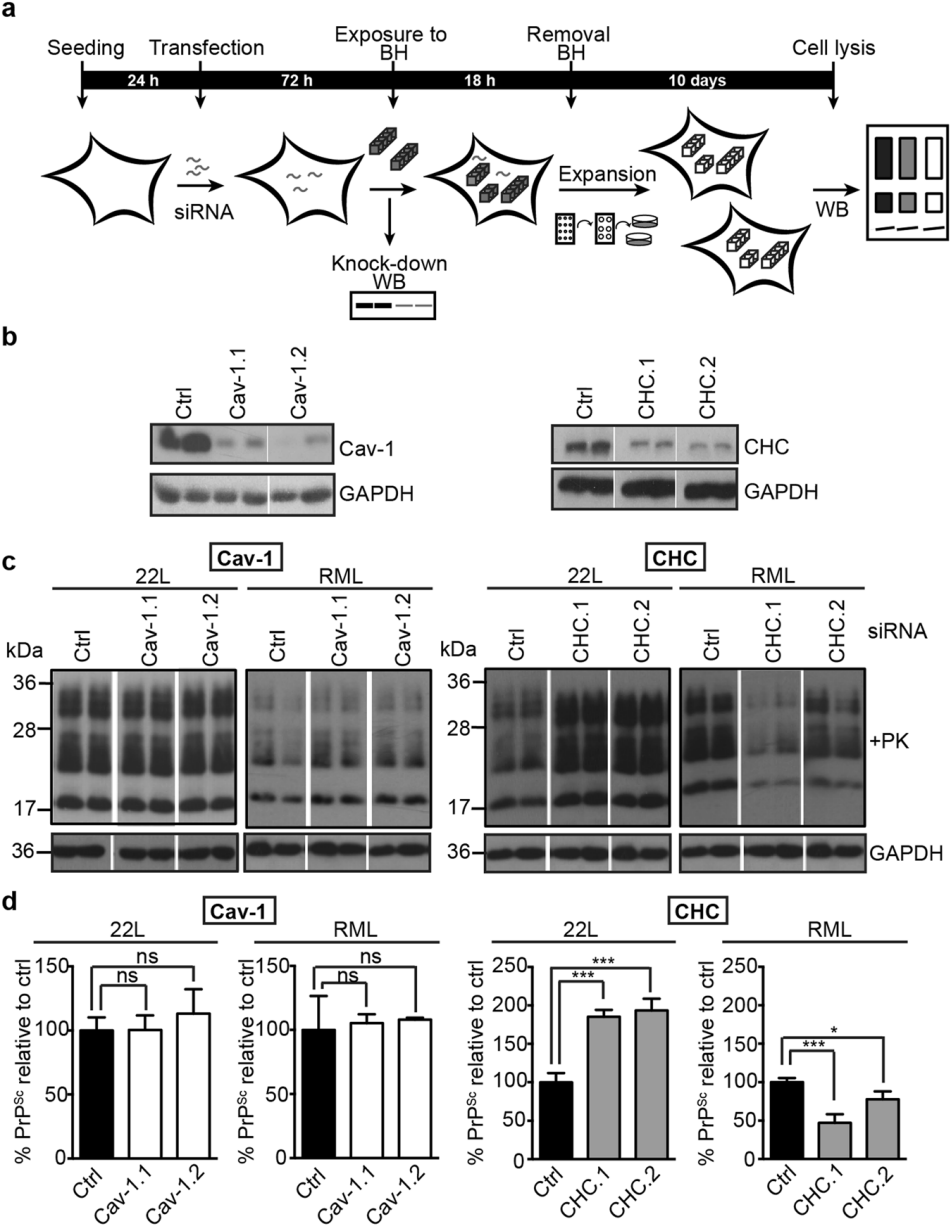
Knock-down of Cav-1 and CHC during acute prion exposure differentially affects establishment of infection. (**a**) Experimental setup. L929 cells were transfected with Cav-1 or CHC siRNAs or non-silencing (Ctrl) siRNA and subsequently exposed to brain homogenates (BH). Cells were cultured for several cell doublings in the absence of BH and tested for PrP^Sc^ content by western blot. (**b**) Efficient knock-down at the time point of infection was confirmed in parallel transfections. Please note that these cells were lysed at 72 h post transfection and not used for the infection experiment. (**c**) Western blot analysis of PrP^Sc^ levels in cells that had been siRNA transfected and passaged 2–3 times post exposure to prions. PrP^Sc^ was detected by mAb 4H11 after PK treatment (+PK), GAPDH protein levels were detected as loading controls in −PK blots. Representative results of one of three independent experiments are shown. For presentation purposes, additional lanes were excized. (**d**) Statistical analysis of PrP^Sc^ signals detected by western blot shown in (**c**). The mean signal value in the sample group transfected with ctrl siRNA was set to 100%. Bars represent mean values ± SD. Statistical analysis was performed using one-way ANOVA with Dunnett’s multiple comparisons test. Signals from four independent transfections/infections were quantified (*p < 0.05, ***p < 0.001, ns = not significant). Experiments were repeated twice with similar results.

The foregoing experiments showed that prion invasion could not be blocked by inhibition of the major endocytic routes. We reasoned that blockage of certain endocytic routes does not inhibit prion uptake but could still traffic PrP^Sc^ to cellular compartments that do not support prion replication. To assess this, knock-down of CHC or Cav-1 was performed at the time point of prion exposure and successful infection was monitored two to three passages post exposure to prion strains (Fig. 5a). At this time point, residual PrP^Sc^ originating from the brain homogenate is diluted out, and PrP^Sc^ detected by western blot represents *de novo* generated abnormal PrP (Supplementary Fig. S8). Interestingly, knock-down of Cav-1 or CHC differentially affected productive prion infections. Both 22L and RML acute infections were insensitive to Cav-1 depletion, as the *de novo* PrP^Sc^ levels were comparable to PrP^Sc^ levels in the non-silencing controls (Fig. 5b, c). By contrast, knock-down of CHC nearly doubled overall levels of newly formed PrP^Sc^ when cells were exposed to 22L (Fig. 5b, c). Importantly, the same treatment led to a strong and significant reduction of PrP^Sc^ in cells infected with RML (Fig. 5b, c). These results suggest that CME is not essential for the establishment of a productive 22L prion infection. By contrast, CME plays a role during the establishment of productive RML infections in L929 cells. Thus, we conclude that the establishment of productive RML and 22L prion infections depends on different endocytic routes.

## Discussion

Prion replication is believed to occur on the cell surface or along the endocytic pathway^11–13^. The role of canonical endocytic routes for the establishment of productive infections by different prion strains is unknown. Here we investigated how manipulation of major endocytic routes during prion uptake, the first days post infection or during chronic infection affect replication of mouse-adapted prion strains 22L and RML in L929 cells. We specifically focused on the role of caveolae-dependent endocytosis and CME for the prion infection process, as both caveolae/rafts and CME are involved in PrP^C^ trafficking and potentially PrP^Sc^ formation^23, 62^. Our comparative analysis of sequential steps of cellular prion infection provides evidence that PrP^Sc^ formation, induced by different prion strains, has different requirements for endocytic trafficking. Specifically, different infection stages appear to be differentially sensitive to disruption of certain endocytic pathways. We cannot exclude that endocytic pathways active in fibroblasts differ from those of susceptible neuronal subsets *in vivo*. It is well established that pathogens exploit different entry pathways in different cell types and cell lines (for review see ref. 63). It is thus possible that prion strains use different cell entry pathways to establish persistent infections in different cell populations. Unfortunately, repetition of these studies in primary neurons is difficult to achieve, as these cultures are hard to transfect, sensitive to environmental perturbations^64^ and also contain a glial subpopulation that increases over time^65^. However, our finding that distinct prion strains depend on different endocytosis routes for productive infection of a given target cell population provides proof-of-concept that the subcellular compartments involved in establishment of infection can fundamentally differ for different strains.

Prion infection *in vitro* includes sequential steps of cell surface attachment, internalization, establishment of productive infection and sustained infection over multiple cell divisions. In analogy to other studies, we find that prions are taken up independent of PrP^C^ expression^33, 54, 57, 58^. The exact mechanism of prion uptake is unknown. In our hands, blockage of caveolae-mediated endocytosis or CME only slightly affected prion internalization. Substantial amounts of PrP^Sc^ were also found internalized by cells with blocked dynamin-dependent endocytosis. These findings suggest that clathrin-, caveolin- and dynamin-dependent pathways are either dispensable for prion uptake by L929 cells or can be bypassed when blocked. Multiple entry routes have already been suggested for prions^15, 66^. The slight but significant differences in strain-specific PrP^Sc^ uptake indicate subtle differences in the entry routes of different strains. Similar subtle differences were recently identified to exist in the uptake of prion strains 22L and 87V in primary neurons^67^. Our data demonstrate that macropinocytosis played a more important role for the uptake of 22L prions compared to RML prions in L929 cells. Interestingly, uptake of 22L prions by primary neurons was shown to be more vulnerable to macropinocytosis inhibitors compared to uptake of 87V^67^. Macropinocytosis has been proposed as a dynamin-independent uptake mechanism for RML prions in another cellular model^62^. It is thus possible that the entry pathway depends on both prion strain and target cell. Macropinocytosis has recently also been reported as an uptake mechanism for other exogenous protein aggregates associated with neurodegenerative diseases^68, 69^. Consistent with our data, macropinocytosis inhibitors reduced superoxide dismutase aggregate uptake by only 20%, arguing that additional pathways might generally participate in the uptake of protein aggregates^68^.

Most studies on prion uptake have not assessed the establishment of infections^22, 70–74^. Importantly, the uptake of PrP^Sc^ does not necessarily lead to persistent infection. Acute PrP^Sc^ formation is even initiated in non-permissive cells or with strains that do not persistently propagate in cell culture^28^. The plasma membrane has been proposed to be the first site of prion conversion^11, 12, 15–17, 75^. While our data do not negate a possible role of the plasma membrane for initial PrP^Sc^ formation, they do point to an involvement of specific endocytic routes for the establishment of productive infections. It is thus possible that initial PrP^Sc^ formation on the cell surface is followed by strain-specific endocytosis events that route the agent through cellular compartments that enable the establishment of an infection. Importantly, different endocytic pathways appear to be required for productive infection by different prion strains. In our hands, caveolae-dependent endocytosis does not play a crucial role for the establishment of 22L or RML prion infections in L929 cells. By contrast, the establishment of an infection with RML prions depends on CME. Surprisingly, impairment of CME significantly increased infection by strain 22L, potentially by increasing the uptake of 22L prions by macropinocytosis. Due to the fact that the majority of cells still take up significant amounts of prions when specific endocytic pathways are blocked, manipulation of endocytosis likely affects downstream events in the establishment of infection. The different infection efficiencies might be the result of prions being routed to subcellular compartments that are more or less favorable for establishment of infection by a particular prion strain. The hypothesis that different prion strains preferentially propagate in different subcompartments is supported by our previous *in vitro* studies^30^ and the here identified strain-specific differences in Optiprep fractionation profiles of PrP^Sc^ propagated in L929 cells. These cellular compartments could for example contain or lack strain-specific cofactors or comprise cellular milieus required for the establishment of productive infections^26, 30, 54, 76^. As prion strains also vary in their resistance to proteolytic degradation^77, 78^, routing to the degradative pathway could have different consequences for different prion strains. However, the fact that inhibition of one endocytic pathway can increase or decrease prion infection depending on the strain argues against the idea that manipulation of endocytic pathways simply increases prion transport for lysosomal degradation. Another possibility is that differences in subcellular trafficking restrict the interaction of prions with potential strain-specific cofactors that are available in different subcellular vesicles. Phospholipids, for example, have been shown to act as cofactors for strain-specific PrP^Sc^ amplification *in vitro* and also vary in relative abundance along the endocytic pathway^79^. Which cellular cofactors catalyse prion strain replication *in vivo* remains to be elucidated.

Curiously, we found that acute and persistent prion infections differed drastically in their dependence on specific endocytic pathways. While caveolae-dependent endocytosis was dispensable during the first days of prion infection, downregulation of this pathway signficantly decreased PrP^Sc^ accumulation in persistently infected cells. This is in line with previous studies that demonstrated that overexpression of Cav-1 in N2a cells persistently infected with RML scrapie led to a more stable infection^10^. Remarkably, no strain-dependent differences were observed for chronic 22L or RML infections. The fact that manipulation of endocytic routes during acute and chronic infections has divergent effects on prion accumulation suggests that the cellular compartments for acute and persistent prion replication differ.

Our data have important implications for the observed strain tropism *in vivo*. Prion strains are characterised by preferential targeting of specific cell types or neuronal subpopulations in the central nervous system^80^. It is possible that uptake by specific entry routes directs prion particles to subcellular compartments that are favorable or less favorable for productive infection by a specific strain. But why would prions be internalized by different routes? Although several putative prion receptors have been described, their role for the establishment of prion infection and their engagement with different prion strains is unclear^22, 23, 73^. Importantly, PrP^Sc^ molecules associated with different strains can be distinguished mainly by their conformation^20^ and not by their association with different cellular proteins^81^. Consequently, conformational differences must impact particle association with cellular receptors or membranes and subsequent intracellular sorting. Endocytic sorting due to pleimorphic morphology of particles has recently been reported for influenza virus^82^. Thus, an interplay between the host cell and the physicochemical properties of prion aggregates could determine the predominant internalization pathway and access to the preferred intracellular compartments. Interestingly, immortalized brain cells have been shown to preferentially internalize PrP^Sc^ subpopulations with comparable sedimentation properties, independent of the strain’s ability to infect cells^83^. Thus, differences in infectivity cannot necessarily be attributed to different PrP^Sc^ subpopulations separable by conventional methods such as sucrose density gradient centrifugation. Rather, the finding that biophysically more stable PrP^Sc^ aggregates associated with a non-productive strain are more efficiently disaggregated intracellularly^83^ is in line with the hypothesis that prion strains are differentially trafficked through the endolysosomal system post internalization. Identification of the subcellular compartments involved in prion strain propagation will be of considerable importance for understanding the pathogenesis associated with distinct prion strains and to unravel potential targets for prion disease intervention.

## Materials and Methods

### Ethics statement

Use of animals in this study was performed in accordance with the recommendations in the Guide for the Care and Use of Laboratory Animals of the National Institutes of Health. The protocol was approved by the Rocky Mountain Laboratories Animal Care and Use Committee (protocol no. 2009-36). The Rocky Mountain Laboratories are fully accredited by the American Association for Laboratory Animal Care. In Germany, animal care was conducted according to German Law. The protocol was approved by the “*Landesamt für Landwirtschaft*, *Lebensmittelsicherheit und Fischerei Mecklenburg-Vorpommern*” (protocol no. LALLF M-V/TSD/7221.3-2.1-012/03).

### Mouse bioassay

A mouse bioassay was performed with inocula prepared from L929 cell cultures exposed to mouse-adapted scrapie strains RML or 22L. Following exposure to prions, cells were split at a ratio of 1:8 every three to four days. After eleven passages cells were subsequently pelleted by centrifugation. Control cells that were exposed to uninfected Mock brain homogenate were collected the same way. Cell pellets were subjected to five freeze-thaw cycles, then adjusted to 1 × 10^7^ cells/ml in Dulbecco’s modified Eagle’s medium (DMEM) (Invitrogen, Karlsruhe, Germany) and passaged through 20 and 24 gauge needles. Additionally, control groups were inoculated with 1% 22L, RML or Mock brain homogenate. A volume of 30 μl of cell extracts (approximately 3 × 10^5^ cells) or brain homogenates were intracerebrally inoculated into C57BL/6 mice (group size: 15). Mice were monitored for TSE-specific clinical symptoms and euthanised upon signs of clinical disease. Incubation times were calculated as the time between the inoculation and the euthanization of the animal.

### Antibodies and siRNA

Mouse monoclonal anti-PrP antibody 4H11 has been described previously^84^. Rabbit polyclonal anti-caveolin-1 (Cav-1) antibody was purchased from Santa Cruz Biotechnologies (Heidelberg, Germany). Mouse monoclonal antibody anti-GAPDH was obtained from Merck Millipore (Darmstadt, Germany) and rabbit polyclonal antibody anti-clathrin heavy chain (CHC) was obtained from Abcam (Cambrige, UK). GFP was indirectly stained with rabbit polyclonal anti-GFP serum purchased from Life Technologies (Darmstadt, Germany).

### Cell culture

Mouse fibroblast cell line L929 (ECACC; L929 (NCTC); #85103115) was purchased from Sigma-Aldrich (Taufkirchen, Germany). Cells were maintained in DMEM containing GlutaMAX and supplemented with 10% fetal calf serum (FCS) and antibiotics. The L929 subclone 15.9, which is highly susceptible to the mouse-adapted scrapie strains 22L and RML, was used for all experiments^24^. This clone was derived by two rounds of limiting dilution cloning and subsequent determination of susceptibility to mouse-adapted prion strains 22L and RML. The selection of susceptible cell clones is a common and necessary method in prion research to achieve higher infection rates *in vitro* ^29, 34–36^.

### Scrapie strains and preparation of brain homogenates

Mouse-adapted scrapie strains 22L and RML were passaged into C57BL/6 mice or the transgenic mouse line Tg(WT-E1) which overexpresses mouse PrP^C^ containing the epitope for the anti-PrP mouse monoclonal antibody 3F4^85^. Mice were specifically infected to produce stocks at the German TSE reference centre. For this purpose, mice inoculated with the particular strain were checked routinely for earliest signs of clinical disease. Animals were sacrificed after reaching a defined humane end point. Brains of the sacrificed mice were routinely checked biochemically to verify the quality and quantity of the deposited PrP^Sc^. Standard operating procedures were implemented and strictly followed for preparation of brain homogenates. Brain homogenates (10% weight/volume) were prepared in OptiMEM medium by use of a dounce homogenizer by 20 strokes and cell debris was removed by low speed centrifugation (870 × g, 4 °C, 5 min). Mock control brain homogenate was prepared from uninfected C57BL/6 mice. Aliquots of brain homogenate were stored at −80 °C. Brain homogenate was chosen as the source for infectious prions as it exhibits increased activity compared to more purified PrP^Sc^ preparations *in vitro* ^54, 86^ and PrP^Sc^ is internalized faster^54, 57^. For uptake and *de novo* infection experiments, brain homogenates from at least three independent brain homogenate preparations were used, yielding similar results.

### siRNA and plasmid transfections

To inhibit CME or caveolae-dependent endocytosis, small interfering RNAs (siRNAs) targeting the clathrin heavy chain (CHC) or Caveolin-1 (Cav-1) were used. SiRNAs for knock-down of Cav-1 (SI00942228 and SI02731813), CHC (SI00953617 and SI00953624) and negative control non-silencing siRNA (AllStars neg. control siRNA, 1027281) were purchased from Qiagen (Hilden, Germany). Knock-down of CHC or Cav-1 was confirmed in every experiment. For perturbation of dynamin-dependent endocytosis, L929 cells were transfected with the dominant negative Dyn2 K44A-GFP construct^59, 60^ or vehicle control pEGFP-N1. Transient transfections were carried out using Lipofectamine 2000 (Invitrogen, Karlsruhe, Germany) and the respective siRNAs according to the manufacturer’s instructions under RNAse free conditions.

### *De novo* prion infection

Prion infections were performed 72 h post transfection. Cells in 24 well plates were exposed to 1% 22L, RML or Mock brain homogenates in DMEM/10% FCS for 5 h, then the brain homogenate was diluted 1:3 with growth medium. The next day the brain homogenate was discarded and the cells were subsequently cultured in fresh medium. Confluent cell monolayers were expanded (passage 1) and cells at passage 2 were tested for PrP^Sc^ content. To control for Cav-1 and CHC knock-down at the time point of infection, additional wells of L929 cells were transfected with siRNA, lysed at the time of infection and analyzed by western blot. Results were confirmed in at least three independent experiments. For binding and uptake experiments after transfection with siRNA or plasmids, cells were extensively rinsed in PBS 18 h post infection and either lysed for western blot analysis or subjected to immunofluorescence staining unless otherwise stated. For inhibition of macropinocytosis, the inhibitor EIPA (80 μM) or DMSO as a solvent control were added to the cells at 37 °C for 1 h prior to addition of brain homogenate.

### Western blot analysis and PK treatment

Cells were lysed and the lysate digested with 20 µg/ml proteinase K (PK) at 37 °C for 30 min. Proteolysis was stopped by adding 0.5 mM Pefabloc. The samples were then precipitated with methanol at −20 °C and PrP^Sc^ detected by an immunoblot assay using antibody 4H11 as described previously^13^. For detection of PrP^C^, Cav-1 and CHC, lysates were not treated with PK but were supplemented with 0.5 mM Pefabloc and precipitated with methanol. Aliquots were analyzed on 12.5% SDS-PAGE gels. PrP^C^ blots were stripped with Re-blot solution (Merck Millipore, Darmstadt, Germany) and re-probed with an antibody directed against GAPDH. For detection of CHC, samples without PK treatment were treated the same way, but analyzed on 7.5% gels. Photodensitometric analysis was performed using the ImageQuant TL (GE Healthcare, Freiburg, Germany) or AIDA Image Analyzer (Raytest, Staubenhardt, Germany). Specific protein bands were normalized to GAPDH bands of the same sample (no PK treatment) and the relative protein levels after knock-down compared to the protein signals in cells treated with non-silencing RNA.

### Flow cytometry analysis

SiRNA-transfected L929 cells were subjected to flow cytometry analysis 72 h post transfection. Cells were detached from dishes using 1 mM EDTA and centrifuged at 320 × g for 2 min. Cells were resuspended in FACS buffer (PBS supplemented with 2.5% FCS and 0.05% sodium azide). For detection of cell surface PrP^C^ all steps were performed on ice. Cells were incubated with anti-PrP monoclonal antibody 4H11 for 45 min, washed and incubated with Cy2-labelled secondary antibody (Dianova, Hamburg, Germany) for 45 min. Cells were then washed and 7-AAD dye (Beckmann Coulter, Krefeld, Germany) was added to visualize dead cells. To detect total (surface and intracellular) PrP^C^ levels, cells were fixed with 4% Roti-Histofix (Roth, Karlsruhe, Germany) at room temperature for 10 min and stained as above at room temperature using FACS buffer supplemented with 0.1% saponin. Data acquisition was performed with a Gallios Flow Cytometer (Beckmann Coulter, Krefeld, Germany) and data were analyzed using the FlowJo software (Tree Star, Inc., Ashland, Oregon, USA). Mean fluorescence intensities (MFIs) of CHC and Cav-1 siRNA treated cells were normalized to MFIs of cells transfected with non-silencing siRNA.

### Flotation assay for detergent-resistant microdomains (DRM) or rafts

Cells were washed twice with MBS pH 6.5 (25 mM MES pH 6.5, 150 mM NaCl) and lysed in 2 ml MES buffer supplemented with 1% Triton-X 100 and complete protease inhibitor cocktail on ice for 30 min. Cell suspensions were homogenized by 20 strokes of a dounce homogenizer and cell debris was pelleted. The supernatant was adjusted to a final concentration of 5% sucrose in MBS. A 5–40% discontinuous sucrose gradient was formed and overlaid with the sample. The samples were ultracentrifuged (189.000 × g, 4 °C) for 18 h in a Beckman type SW41Ti rotor. Fractions (1 ml) were collected from the top to the bottom of the ultracentrifuge tube and proteins were precipitated with 5 volumes of methanol. After centrifugation at 2.600 × g for 25 min, pellets were resuspended and equal volumes were analyzed by western blot analysis. For PrP^Sc^ detection, methanol precipitated proteins were resuspended in PBS and subjected to 50 μg/ml proteinase K for 45 min at 37 °C. Proteolysis was terminated by addtion of Pefabloc and proteins were separated as above.

### Surface biotinylation assay

L929 cells were plated and transiently transfected with siRNA as described previously. At 72 h post transfection, cells were rinsed with ice cold PBS and biotinylated with 250 mg/ml Sulfo-NHS-LC-Biotin (Thermo Scientific, Schwerte, Germany) on ice for 20 min. Cells were subsequently washed with cold PBS, incubated with 20 mM Glycin/50 mM NH_4_Cl on ice to quench the biotinylation reaction and rinsed again with ice cold PBS. Cultures were chased at 37 °C for 60 min to allow internalization of PrP^C^. Cells were subsequently incubated with or without trypsin on ice for 10 min. Proteolysis was terminated by addition of soybean trypsin inhibitor. After lysis of cells, PrP^C^ was immunoprecipitated using the monoclonal antibody 4H11 at 4 °C over night and subjected to SDS-PAGE followed by immunoblot. Biotinylated PrP^C^ was detected using horseradish peroxidase- (HRP-) conjugated streptavidin and signals were analyzed densitometrically. The amount of internalized PrP^C^ (+Trypsin) was expressed as a percentage of the amount of total biotinlytated PrP^C^ detected in control cells without Trypsin treatment (−Trypsin). The mean signal value in this sample group was set to 100%.

### Transferrin and cholera toxin uptake assays

L929 cells were plated on glass bottom dishes (35 mm, MatTek *In Vitro* Life Science Laboratories, Bratislava, Slovak Republic) and transfected with siRNA as described previously. Uptake experiments were performed 72 h post transfection. For transferrin (Tfn) uptake experiments cells were rinsed once with PBS and incubated in serum-free DMEM containing 40 µg/ml transferrin conjugated to Alexa Fluor 488 (Tfn-AF488, Molecular Probes, Thermo Fisher, Darmstadt, Germany) at 37 °C for 30 min. After incubation, cells were washed twice with PBS and living cells were imaged by confocal microscopy. To follow the uptake of cholera toxin subunit B (CtxB) upon Cav-1 silencing, cells were washed in PBS supplemented with 10 mM HEPES and incubated with 7.5 µg/ml Alexa Fluor 647-labelled cholera toxin subunit B (CtxB-AF647, Molecular Probes) at 10 °C for 30 min. Buffer was replaced with fresh HEPES-PBS and cells were further incubated at 37 °C for 40 min prior to a second extensive rinsing. Cells were subsequently imaged by confocal microscopy for CtxB uptake. Fluorescence intensities in at least 50 cells per dish were analyzed using ImageJ software (http://imagej.nih.gov/ij/). The relative uptake of Tfn and CtxB was expressed as a percentage of the signal intensities in non-silencing siRNA transfected control cells.

### Fluid-phase uptake

The fluid-phase uptake assay was performed 72 h post transfection except for infection experiments where, after inhibition of macropinocytosis, cells were analyzed for FITC-dextran uptake at the time point of infection. Cells were incubated with 0.5 mg/ml 70 kDa FITC-dextran for 30 min, then placed at 4 °C and washed twice with ice cold PBS and once with 0.1 M sodium acetate (pH 5.5)/0.05 M NaCl to remove surface-bound dextran. Cells were detached with 0.25% Trypsin-EDTA on ice for 25 min and transferred into PBS containing 7% FCS to inhibit proteolysis. After low-speed centrifugation (320 × g, 2 min) cell pellets were resuspended in 3.7% paraformaldehyde (PFA) (pH 7.4), fixed on ice for 20 min and washed again. Cells in PBS were analyzed by flow cytometry. Cells treated with the macropinocytosis inhibitor EIPA (80 μM) or DMSO at 37 °C for 1 h prior to addition of FITC-dextran were used as controls for impaired fluid-phase endocytosis. As a control for the complete removal of membrane-bound FITC-dextran, additional cultures were kept at 4 °C and analyzed in parallel in every experiment.

### Confocal laser scanning microscopy

Cells were prepared for immunofluorescence analysis as described previously^78^, with slight modifications. Briefly, cells plated on coverslips were fixed using 3.7% PFA (pH 7.4), permeabilized (0.1% Triton X-100) and proteins were denatured using 6 M guanidinium hydrochloride. Samples were rinsed with PBS, blocked in 0.2% gelatine and incubated for 2 h with primary antibody diluted in blocking solution. After three washing steps in PBS, cells were incubated for 1 h with Alexa Fluor 488- or Cy3-conjugated secondary antisera (Life Technolgies, Darmstadt, Germany; Dianova, Hamburg, Germany) and nuclei stained with Hoechst DNA staining dye (Sigma, Taufkirchen, Germany). When number and size of intracellular PrP^Sc^ puncta were analyzed, cells were also stained with HCS CellMask Blue stain (Life Technolgies, Darmstadt, Germany) according to the manufacturer’s protocol. After washing, slides were mounted in Aqua-Poly/Mount (Polysciences, Eppelheim, Germany). Confocal laser scanning microscopy was performed using a LSM 700 laser scanning microscope (Zeiss, Jena, Germany). All samples within one experiment were imaged with the same acquisition and filter settings.

### Quantification of intracellular puncta after exposure to scrapie brain homogenates

Confocal images were acquired using the LSM 700 laser scanning microscope with a 63x objective. An image analysis routine was developed using the CellProfiler cell image analysis software^87^ to determine the number and size of intracellular PrP^Sc^ puncta per cell. Segmentation of images was performed by adjustment of CellProfiler’s object identification modules. Nuclei of single cells were identified as primary objects and the algorithm was adjusted *via* morphology characteristics and intensity levels of Hoechst. To determine cell boundaries, differences in the intensity levels of CellMask Blue stain were detected in the same channel as Hoechst. Cell boundaries and corresponding nuclei were used to identify single cells. Cells that were only partly imaged (cells at the boundaries of the image) were excluded from the analysis. PrP^Sc^ puncta were identified as secondary objects by Cy3 fluorescence determination. The module was carefully adjusted to morphology and intensity levels and identified puncta were linked to host cells. Mock infected cells processed in parallel were used as controls for the specific detection of PrP^Sc^ and to exclude PrP^C^ background staining. The number of cells harboring intracellular puncta and cells without puncta were determined, and cells were grouped as PrP^Sc^ positive or PrP^Sc^ negative. The percentage of cells with intracellular puncta to total number of cells was calculated using Excel. The size of puncta was measured as the pixel area per identified object and consecutively translated in μm². On average 100 cells per group were analyzed in RNAi and macropinocytosis experiments. In uptake experiments following inhibition of dynamin, at least 40 cells per group were analyzed.

### Statistical analysis

Results obtained from image analysis were tested for normal distribution and comparisons were made with non-parametric Mann-Whitney test or the unpaired two-tailed Student’s t test. Significant differences between individual biological replicates in western blots were determined using the unpaired two-tailed Student’s t-test for single comparisons or one-way ANOVA with Dunnett’s multiple comparison test using Graph Pad Prism software. P values < 0.05 were considered significant. Experiments were repeated at least twice with consistent results.

### Data availability

All data generated or analyzed during this study are included in this published article (and its Supplementary Information files).

## Electronic supplementary material

Supplementary Information
