# Supplementary material for: Prion strains depend on different endocytic routes for productive infection
Source: Sci Rep. 2017 Jul 31;7:6923. doi: 10.1038/s41598-017-07260-2 (PMC5537368; doi:10.1038/s41598-017-07260-2)

Supplementary Information

Prion strains depend on different endocytic routes for productive infection

Andrea Fehlinger[1], Hanna Wolf[1], André Hossinger[1], Yvonne Dürnberger[1], Catharina Pleschka[1], Katrin Riemschoss[1], Shu Liu[1], Romina Bester[2], Lydia Paulsen[1], Suzette A. Priola[3], Martin H. Groschup[4], Hermann M. Schätzl[5] and Ina Vorberg[1, 6*]

1 Deutsches Zentrum für Neurodegenerative Erkrankungen e.V., Sigmund-Freud-Strasse 27, 53127 Bonn, Germany2 Institut für Virologie, Technische Universität München, 81675 München, Germany

3 Laboratory of Persistent Viral Diseases, Rocky Mountain Laboratories, National Institute for Allergy and Infectious Diseases, National Institutes of Health, Hamilton MT 59840, USA

4 Friedrich-Loeffler-Institut, Institute of Novel and Emerging Infectious Diseases, 17493 Greifswald-Insel Riems, Germany

5 Dept. of Comparative Biology & Experimental Medicine, University of Calgary, Calgary, AB T2N 4Z6, Canada

6 Department of Neurology, Rheinische Friedrich-Wilhelms-Universität, 53127 Bonn, Germany

*Corresponding author: E-mail: ina.vorberg@dzne.de (IV)

Supplementary Table. L929^{RML} and L929^{22L} produce infectious prions as assessed by mouse bioassay.

To confirm that L929 cells produce prion infectivity, mouse bioassays were performed in C57BL/6 mice. Mice were inoculated intracerebally with extracts from RML or 22L prion infected L929 cells or from uninfected Mock cells. Prior to the bioassay, cells had been passaged 11 times at a ratio of 1:8. By the time of inoculation, the actual infectious dose present in the inoculum would have been diluted out. Prion infected brain homogenates used to infect the cells were injected in parallel as positive controls. The table displays the attack rate and mean incubation times.

Supplementary Fig. S1. Knock-down of Cav-1 and CHC lead to functional impairment of caveolae- or clathrin-mediated endocytosis. L929 cells were transfected with non-silencing siRNA or siRNA directed against Cav-1 (**a**) or CHC (**b**) and cultured for 72 h. **a., c.** Control and Cav-1 or CHC siRNA treated cells were incubated with fluorophor-labelled Choleratoxin B (CTxB-AF647; red) or Transferrin (Tfn-AF488; green). Scale bars: 5 µm. **b.**, **d.** Graphs show the relative CTxB and Tfn uptake normalised to control cells. Bars represent mean values ± SD. Statistical analysis was performed using the unpaired two-tailed t-test. Significant changes are indicated by asterisks (***p < 0.001).

Supplementary Fig. S2. Knock-down of CHC and Cav-1 by two indendent siRNAs. For determination of knock-down efficiencies, L929 cells were transfected with the respective siRNAs or non silencing control siRNA. Protein levels were assessed by western blot 72 h post transfection. Each siRNA was tested in 3-4 independent transfections in uninfected and prion-infected cells. Bars represent mean values ± SD. Statistical analysis was performed using the unpaired two-tailed t-test. Significant changes are indicated by asterisks (****p < 0.0001; ****p < 0.001).

Supplementary Fig. S3. Genistein treatment reduces PrP^{Sc} levels in L929 cells persistently infected with prion strains 22L and RML. a. L929 cells were treated with 100 µM genistein or DMSO solvent (ctrl) for 72 h, and CTxB uptake was examined in living cells by confocal laser

scanning microscopy. Scale bar: 5 µm. **b.** Western blot analysis of L929 cells persistently infected with 22L or RML prions treated for 72 h with genistein or DMSO solvent (ctrl). PrP^{Sc} was detected in lysates treated with PK (+PK) using mAB anti-PrP 4H11. Blots showing total PrP (-PK) were reprobed for GAPDH as a loading control. Additional lanes were excised for presentation purposes. **c.** Quantitative comparison of PrP^{Sc} levels in control and genistein treated cells. The amount of PrP^{Sc} present in lysates of cells treated with genistein was compared to the PrP^{Sc} amount present in solvent treated control cells (set to 100 %). Bars represent mean values ± SD. Statistical analysis was performed using One-way ANOVA with Dunnett's multiple comparisons test. Significant changes are indicated by asterisks (n = 3; ****$p < 0.001$, ns = not significant).

Supplementary Fig. S4. PrP^{C} tagging reveals absence of *de novo* formed PrP^{Sc} 18 h post exposure to brain homogenate. a. Schematic illustration of the experimental setup. Tagged mouse PrP^{C} (3F4-PrP^{C}) was used to discriminate newly synthesised from inocula-derived PrP^{Sc}. Endogenous, *de novo* generated 3F4-PrP^{Sc} can be distinguished from PrP^{Sc} present in the inoculum by the epitope specific antibody 3F4 (BH: Brain homogenate). **b.** Western blot analysis of L929 cells expressing 3F4-PrP (3F4-L929) exposed to brain homogenate from mice infected with scrapie strain 22L (lanes 4 and 5). Cells were rinsed extensively and lysed 18 h post exposure to BH. Samples were treated with PK (+PK) and PrP^{Sc} was detected by western blot using mouse monoclonal antibody 4H11 (upper panel) or 3F4 antibody for specific detection of *de novo* generated PrP^{Sc} (lower panel). As controls, chronically 22L infected L929 cells ($L929^{22L}$, lane 1) and L929 cells expressing 3F4-PrP which had been persistently infected with mouse scrapie strain 22L (3F4-$L929^{22L}$, lane 2) were used. Lysate from cells exposed to Mock infected brain homogenate (BH^{Mock}) was used to control for the specific detection of PrP^{Sc} after PK treatment (lane 3). Note that the PrP^{Sc} signal produced in infected cells differs from the one originating from the inoculum (compare lanes 1, 2 to 4, 5 in upper panel).

Supplementary Fig. S5. Uptake of PrP^{Sc} in L929 cells is independent of PrP^{C} expression. a. Graphical illustration of the experimental setup. BH: Brain homogenate, WB: Western blot, **b.** L929 cells transfected with siRNA against PrP^{C} were lysed 72 h post transfection to test for PrP^{C} expression

by WB. Anti-PrP antibody: 4H11. **c.** Expression level of PrP^C relative to control as assessed by WB. Three independent transfections were performed. **d.** Uptake of 22L or RML PrP^{Sc} in siRNA transfected cells was monitored by immunoflourescence staining 18 h post exposure to prions. PrP^{Sc} was detected with mAb 4H11 (red) after denaturation with GdnHCl. Nuclei were stained with Hoechst (blue). Cells exposed to Mock brain homogenate served as controls. Every sample group was imaged with identical settings. Scale bar: 10 µm. **e.** Analysis of cells with PrP^{Sc} signal. A total number of at least 100 cells was scored. Three independent experiments were performed.

Supplementary Fig. S6. Uptake of PrP^{Sc} in L929 cells is dynamin-2-independent. a. Experimental setup to analyse fluorophore-labelled transferrin uptake by cells expressing the dominant negative Dyn K44A-GFP mutant. **b.** Expression of dominant-negative dynamin-2 mutant K44A in L929 cells inhibits uptake of transferrin. L929 cells were transfected with a construct coding for Dyn K44A-GFP or the vector alone as vehicle control (pEGFP-N1). 72 h post transfection, cells were incubated with Alexa Fluor 568-labelled transferrin (Tfn-AF568) as described in (**Fig. 1d**) and imaged by confocal microscopy. Asterisks indicate transfected cells. Scale bar: 20 µm. **c.** Experimental setup to analyze PrP^{Sc} uptake upon expression of the dominant negative Dyn2 K44A-GFP mutant. Uninfected L929 cells were transfected with the Dyn K44A-GFP construct or the vector alone as vehicle control and exposed to brain homogenate (BH) 72 h post transfection. Uptake of PrP^{Sc} was monitored by confocal microscopy analysis. **d.** Transfected L929 cells exposed to Mock, 22L or RML brain homogenate. Cells were rinsed extensively and fixed for subsequent immunofluorescence staining following GdnHCl treatment. PrP^{Sc} was detected using mAb 4H11 (red). GFP was detected using anti-GFP antibody (green) because of loss of GFP autofluorescence by protein denaturation. Nuclei were stained with Hoechst (blue). Note that under the conditions used, PrP^C was not detected in uninfected Mock cells. Scale bar: 5 µm.

Supplementary Fig. S7. Silencing of clathrin heavy chain expression increases macropinocytosis. a. Experimental setup to demonstrate the effect of siRNA mediated knock-down of CHC on macropinocytosis. L929 cells were transfected with siRNA against Cav-1, CHC or with non-silencing siRNA. Cells were subsequently incubated with FITC-dextran, fixed and analysed by flow cytometry.

As a positive control for inhibition of macropinocytosis, cells were treated with EIPA for 1 h prior to incubation with labelled dextran. **b.** MFIs of CHC or Cav-1 siRNA transfected or EIPA treated cells were normalised to the MFIs of the corresponding control cells set to 100%. Bars represent mean values ± SD. Statistical analysis was performed using the unpaired two-tailed t-test. Significant changes are indicated by asterisks (n = 3, ***$p \leq 0.001$, ns = not significant).

Supplementary Fig. S8. No residual 22L PrP^{Sc} is detectable in passages 2 and 5 post infection. Western blot analysis of L929 cells exposed to 22L infected brain homogenate from mice expressing 3F4-PrP (3F4-BH^{22L}) which accumulate 3F4-tagged PrP^{Sc}. Cells were lysed in passage 2 (upper panel) and passage 5 (lower panel) post infection and PK treated samples were analysed for PrP^{Sc} by western blot analysis using mAb 3F4 (**a**) or 4H11 (**b**). Lanes 1 - 4 show cell lysates from independent infections. PK treated lysates from cells exposed to brain homogenate from Mock infected 3F4-PrP mice (3F4-BH^{Mock}) were used as controls (lane 5). As positive controls for the antibodies, L929 cells persistently infected with 22L (L929^{22L}, no 3F4-tagged PrP^{Sc} should be detected, lane 6) and 22L infected L929 cells expressing 3F4-PrP (3F4-L929^{22L}, lane 7) were used.

Supplementary table

Inoculum	Incidence[a]	Incubation time (days)[b]
Cell extracts		
L929^{RML}	15/15	152 ± 14.0
L929^{22L}	15/15	172 ± 16.1
L929^{Mock}	0/14*	-
Brain homogenates		
RML	14/14	132 ± 9.5
22L	13/13	126 ± 5.7
MOCK	0/14	-

[a] Number of sacrificed mice with TSE symptoms/infected mice

[b] Mean incubation time ± SD days post infection

* One mice died of unknown cause throughout the course of the experiment.

Supplementary Figure S1

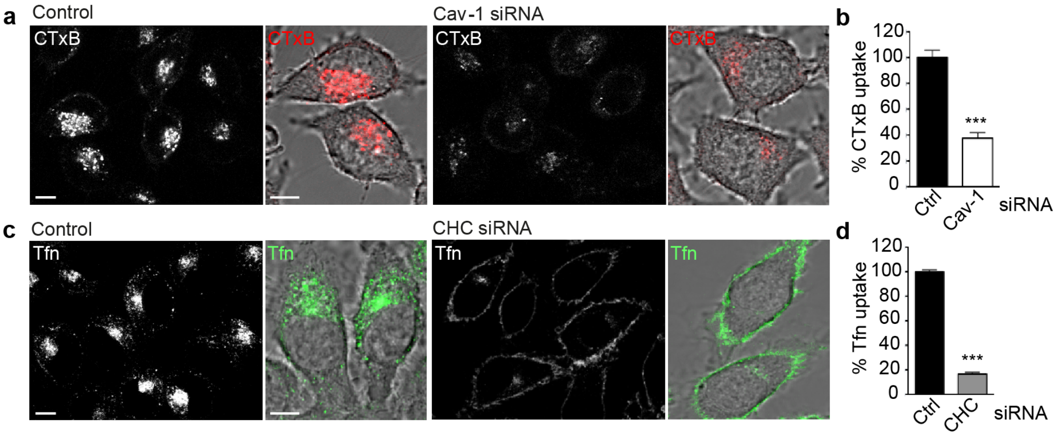

Supplementary Figure S2

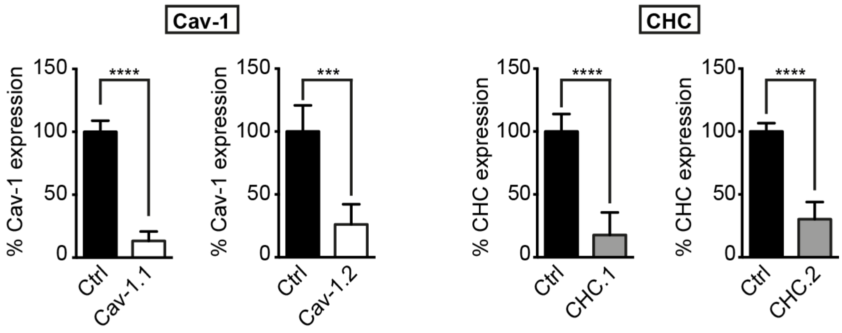

Supplementary Figure S3

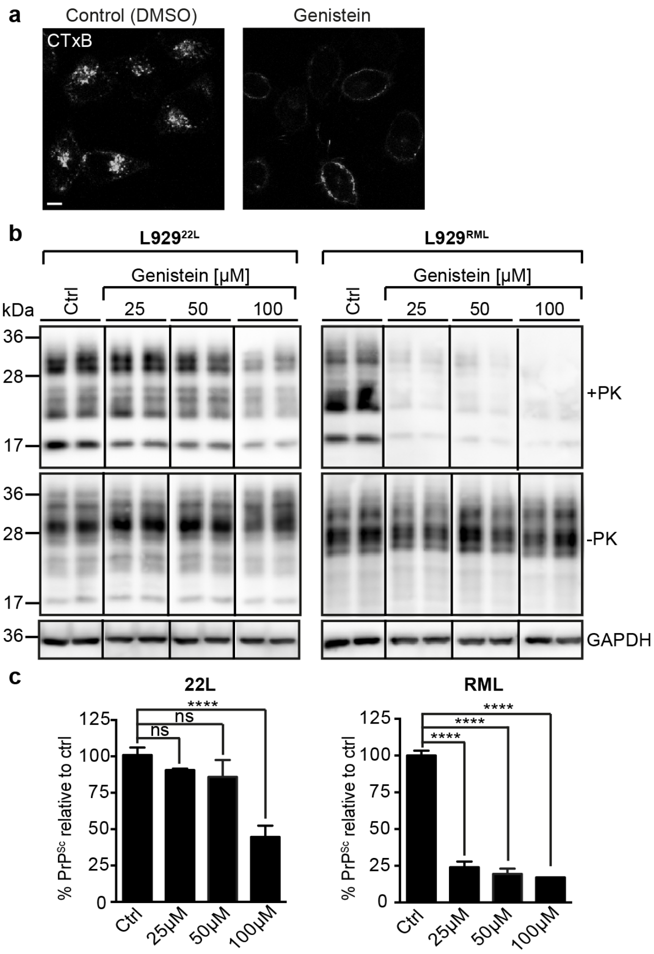

Supplementrary Figure S4

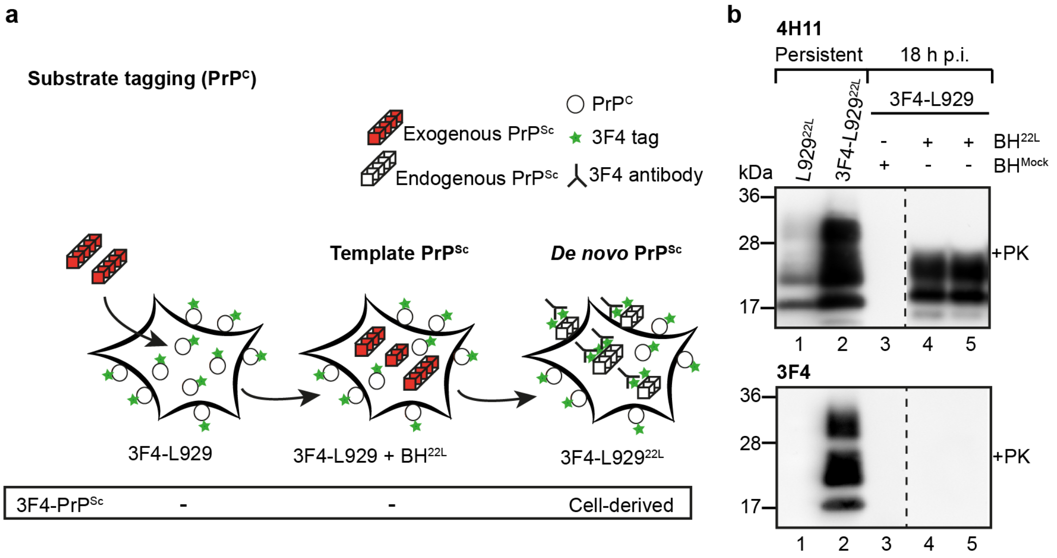

Supplementary Figure 5

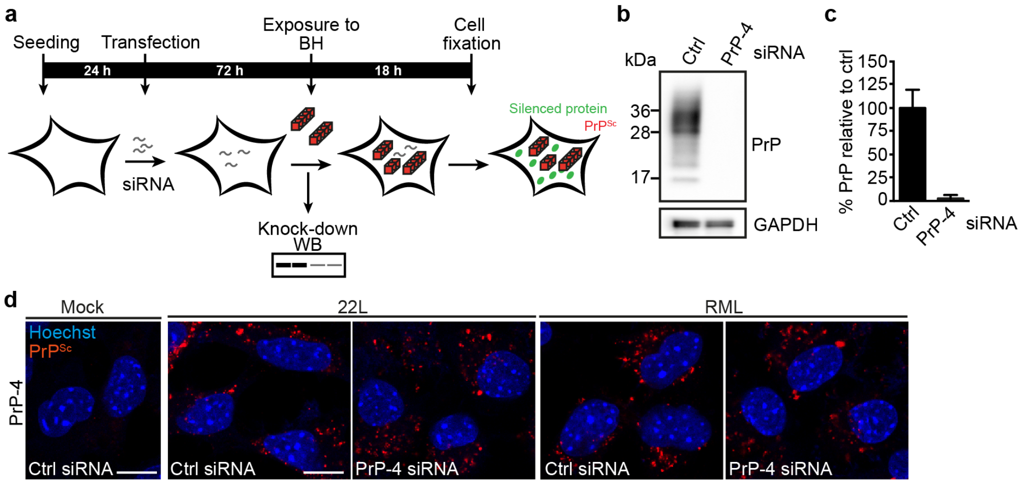

e

% cells with uptake of PrP^{Sc}		
	22L	RML
Ctrl siRNA	96.4 ± 2.1	89.3 ± 3.8
PrP-4 siRNA	98.9 ± 1.1	93.6 ± 4.8

Supplementary Figure 6

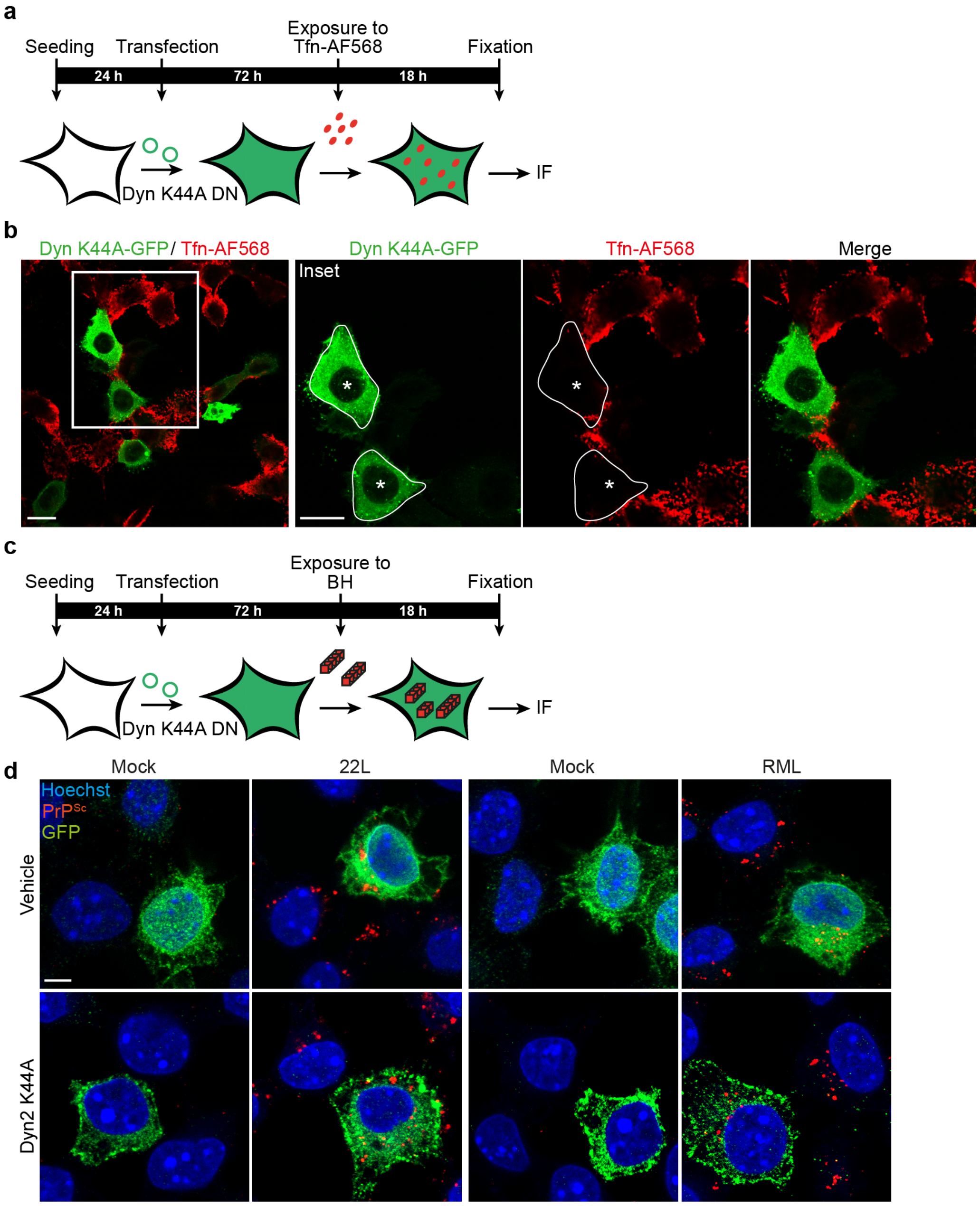

Supplementary Figure S7

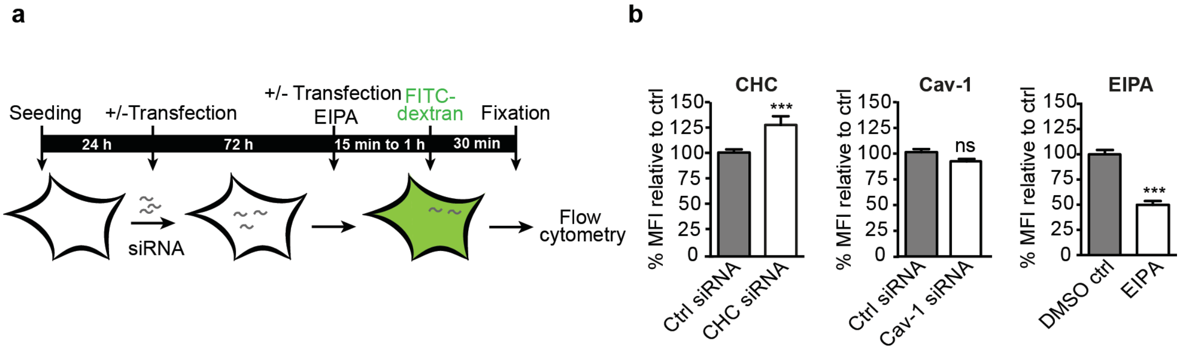

Supplementary Figure 8

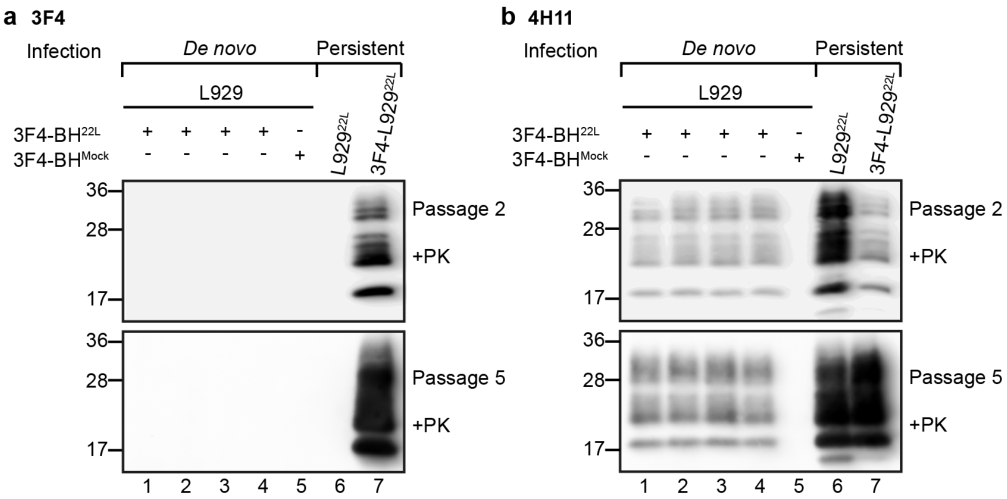

Supplement: Supplementary file 1 — Supplementary Information [file 41598_2017_7260_MOESM1_ESM.pdf]
